# Development of the Greifswald questionnaire for the measurement of interprofessional attitudes

**DOI:** 10.3205/zma001300

**Published:** 2020-02-17

**Authors:** Sandra Lange, Maud Partecke, Konrad Meissner, Ulrike Heß, Anette Hiemisch

**Affiliations:** 1 Universität Greifswald, Institut für Psychologie, Greifswald, Germany; 2 Universitätsmedizin Greifswald, Körperschaft des öffentlichen Rechts, Klinik für Anästhesiologie, Intensiv-, Notfall- und Schmerzmedizin, Greifswald, Germany; 3 Universitätsmedizin Greifswald, Körperschaft des öffentlichen Rechts, Geschäftsbereich Pflege, Praxisanleitung, Greifswald, Germany

**Keywords:** interprofessional education, questionnaire development, interprofessional attitudes, attitude measurement

## Abstract

**Introduction: **The implementation of interprofessional education (IPE) could be a potential approach to dealing with increasing complexity in health care. And thus, encouraging interprofessional collaborations to minimize errors in patient care. However, empirical evidence regarding the effectiveness of IPE is inconclusive. One reason for this is a lack of valid and reliable evaluation instruments. This study aims to illustrate the first steps of the development and validation of a German evaluation instrument for the measurement of interprofessional attitudes.

**Methods: **To achieve high psychometric quality, we first selected relevant attitude dimensions and specified criteria for the wording of the items. The a priori developed factor structure was evaluated via factor analysis and the internal consistencies of the scales were analysed in a sample of medical students and nursing trainees, both participants of an IPE course (n=338). Stability was evaluated in an additional sample of nursing trainees (n=14).

**Results: **The Factor analysis revealed three dimensions. Whereby, the two factors “Relevance of learning interprofessional communicational techniques” (German: Wichtigkeit Techniken interprofessioneller Kommunikation zu lernen) (α=.85) and “Doubts, dismissal and perceived barriers” (German: Zweifel, Ablehnung und wahrgenommene Barrieren) (α=.73) revealed good to acceptable internal consistency. Third-factor “Attitude towards another profession” (German: Einstellung zur anderen Berufsgruppe) (α=.62) remained below a desired internal consistency of α>.70. Factors “Doubts, dismissal and perceived barriers“, as well as “Attitude towards another profession” showed sufficient stability for pre-/post-measurements.

**Discussion: **The Greifswald Questionnaire for the Measurement of Interprofessional Attitudes is the first version of a three-dimensional tool to evaluate IPE in German-speaking countries. Results showed insufficient item difficulty in the tested sample, which resulted in an insufficient internal consistency, and retest reliability for some factors. Further studies are required to investigate item difficulty, internal consistency and retest reliability in a postgraduate sample.

## Zusammenfassung

**Einleitung: **In einem komplexer werdenden Gesundheitssystem sind Maßnahmen nötig, um Kompetenzen der interprofessionellen Zusammenarbeit zu fördern und somit das Fehlerpotential bei der Versorgung von Patienten zu minimieren. Eine Maßnahme, um dies zu erreichen, könnte die Integration von interprofessionellem Lernen (IPL) in die Ausbildung von Gesundheitsberufen sein. Es fehlen bisher allerdings belastbare Befunde, die die Wirksamkeit von IPL belegen. Eine Ursache hierfür ist die geringe Validität und Reliabilität verfügbarer Evaluationsinstrumente.

Die vorliegende Publikation beschreibt erste Schritte der Validierung eines deutschsprachigen Evaluationsinstrumentes zur Messung interprofessioneller Einstellungen.

**Methoden:** Um eine möglichste hohe Validität und Reliabilität zu erreichen, wurden zunächst geeignete Fragebogendimensionen ausgewählt sowie methodische Kriterien für die Itemkonstruktion festgelegt. An einer Stichprobe von Studierenden der Humanmedizin sowie Auszubildenden der Gesundheits- und Krankenpflege, die gemeinsam an einem IPL-Kurs teilnahmen, wurden die a priori festgelegten Skalen faktorenanalytisch überprüft und hinsichtlich ihrer internen Konsistenz analysiert (N=338). Die Stabilität der Dimensionen wurde an einer weiteren Stichprobe von 14 Auszubildenden der Gesundheits- und Krankenpflege untersucht.

**Ergebnisse:** Es wurden drei Einstellungsdimensionen extrahiert. Die Dimensionen Wichtigkeit Techniken interprofessioneller Kommunikation zu lernen (α=.85) sowie Zweifel, Ablehnung und wahrgenommene Barrieren (α=.73) sind gut bis akzeptabel intern konsistent. Unterhalb eines angestrebten Niveaus von α>.7 blieb die Dimension Einstellung zur anderen Berufsgruppe (α=.62). Eine ausreichende Stabilität für Prä-/Post-Erhebungen konnte für die Dimensionen Zweifel, Ablehnung und wahrgenommene Barrieren und Einstellung zur anderen Berufsgruppe nachgewiesen werden.

**Diskussion: **Mit dem Greifswalder Fragebogen zur Messung interprofessioneller Einstellungen (GreiFMIE) liegt die erste Version eines dreifaktoriellen Fragebogens zur Evaluation von IPL vor. In der untersuchten Stichprobe erwies sich die Itemschwierigkeit als zu leicht, wodurch sich für einige Fragebogen-Dimensionen nicht ausreichende interne Konsistenzen und Retest-Reliabilitäten ergaben. Weiterführende Untersuchungen sollen zeigen, ob eine höhere Itemschwierigkeit bei postgraduierten Gesundheitsberufen vorliegt.

## 1. Introduction

First-rate healthcare requires an effective collaboration between different health care professions. Thus, instead of first meeting at the workplace, the different professions should be educated together [1]. Consequently, over the last 20 years, a variety of different course concepts for interprofessional education (IPE) were established internationally (see [2], [3], [4], [5]). However, empirical evidence regarding the effectiveness of IPE is inconclusive [6]. The reasons range from the heterogeneity of the course concepts over unsatisfactory evaluation designs to a lack of reliable and valid instruments to measure IPE outcomes (see [7], [8]).

The present study addresses the latter point. We present the Greifswalder Questionnaire for the Measurement of Interprofessional Attitudes (GreifMIE). A questionnaire, designed to reliably and validly assess attitudes that are relevant for successful collaborative practice and can be modified by IPE-courses. 

In the following, we first discuss methodological requirements for IPE evaluation instruments, then illustrate how these requirements were implemented throughout the development of the GreifMIE, and finally report first results regarding its psychometric properties. 

### 1.1. IPE outcomes 

The first step in developing an instrument for IPE evaluation is to identify the level and the characteristics of the expected outcomes. We applied the following classifications, specifically developed for IPE evaluation [9], whereby meeting the outcomes on a lower level is a pre-condition for the attainment of higher-level outcomes.

- **Level 1:** Learners’ reactions (e.g. satisfaction with the course)
- **Level 2a:** Modification of attitudes/perceptions
- **Level 2b:** Acquisition of knowledge/skills
- **Level 3:** Change in behaviour
- **Level 4a:** Change in organizational practice
- **Level 4b: **Benefits to patients/clients

For the assessment of Level 1, a variety of questionnaires for student’s course evaluation are available at most universities. While sometimes an adaptation to the specific features of a course may be useful, there is no demand for the development of a new IPE instrument for level 1. In contrast, the evaluation of the higher levels requires specialized instruments. The GreiFMIE focusses on Level 2a and aims to assess outcomes which can validly be assessed via self-report.

#### 1.2. Measuring IPE outcomes via self-reports

Self-report measurement is a typical way of data collection in the social sciences [10]. To gain access to internal states such as attitudes or the quality of emotions, it may provide the exclusive way to gather information. However, depending on the content of the self-report, the measurements can be prone to different types of error. Self-reports of internal states, and if carefully prompted also self-reports of behaviour are generally valid (see [11], [12]). By contrast, self-reported competencies (level 2b: knowledge and skills) are rather error-prone. 

Subsequently, we discuss requirements of measurement of IPE outcomes on the levels 2b, 3 as well as 4a and 4b. Thereby illustrating why we decided against the measurement of these levels.

##### 1.2.1. Measuring level 2b

Competencies bridge the gap between knowledge and a successful action [13]. Successful interprofessional communication requires not only knowledge about the techniques of interprofessional communication, but also the skills to apply them. Furthermore, the standard against which the quality of the skills are measured is the effect a person’s communication has on the team members. To realistically apply this standard, the person needs to understand the indicators of successful communication and has to be able to recognize them in others. Thus, a pre-requirement for valid self-assessment of competence is that a person already possesses the competence, at least to a certain degree [14]. Consequently, self-assessments of relatively unskilled persons are more flawed than those of skilled persons [15]. This systematic error renders self-assessed competencies unsuitable for course evaluation [16].

##### 1.2.2. Measuring level 3

Ultimately, IPE is effective when it improves the participants' work behaviour. Hence, behavioural changes are the core dimensions of the IPE evaluation. However, in the case of pre-post comparisons, the assessment of behaviour poses a problem, because the outcomes measured in the pre-test, are only just acquired during the course. As the GreiFMIE is designed for pre-post comparisons as well as comparisons between different healthcare professions, regardless of whether they have participated in an IPE course, we decided against the measurement of behaviour. 

##### 1.2.3. Measuring level 4a and 4b

For the evaluation of Level 4 (change in practice and benefit to patients) it should be demonstrated that the skills taught in IPE courses are applied during the daily work routine, and additionally have a positive impact on patient care. Only in the first case (work behaviour) self-reports provide valid information. Nevertheless, items about attitudes are often applied to evaluate level 4 (see [17], [18]), even though attitudes of healthcare professionals regarding the outcomes for patients are not identical with the actual outcomes – such as the reduction of treatment errors.

For the above reasons, the GreiFMIE aims at the evaluation of level 2a. Specifically, it assesses IPE-relevant attitudes and perceptions before or after an IPE-intervention via self-reports.

#### 1.3. Questionnaire design

The meaning of evaluation results for medical education depends on their validity [15], [19], which in turn is based on psychometric properties, such as reliability and the overall psychometric quality of an instrument and its items. 

In a review, Oates and Davidson [7] found 140 English instruments for the measurement of IPE outcomes. For 44 of these, insufficient information about reliability and validity were provided. Altogether, only 9 questionnaires qualified for further analysis, and even for those, the psychometric integrity was limited. Hence, we decided to develop a new questionnaire to explicitly address the discussed problems. In the first phase of the development, we focused on the internal structure of the questionnaire and guidelines for wording of the items, to achieve sufficient validity, and internal consistency of the scales. A detailed discussion of the first two points is presented below, while the internal consistencies are discussed in the method section.

#### 1.4. Internal structure

In 2014, a project on “increasing patient safety by integrating human factor training in the training of health professions” was initiated by the Department of Anesthesiology and the vocational training school at the Department of Medicine at the University of Greifswald [20]. Pre-qualification health professional students (mostly doctors and nurses) were educated together in the field of emergency medicine. This was the starting point for the development of the GreiFMIE. 

IPE frameworks seemed a logical source for determining the attitude dimensions to be included in the GreiFMIE [21]. However, these frameworks are not theories of IPE, but guidelines, specifying IPE relevant skills, often against the background of national health care systems. No binding framework is available for German-speaking countries. Consequently, as a theoretical guideline for instrument design, these frameworks are suitable only to a limited extent. Likewise, basing the development on the educational concept of the project was thought to result in a limited area of application. 

Thus, in order to determine IPE relevant attitude dimensions applicable for pre-post comparisons of different course concepts we employed two sources:

1. The expert knowledge of the course coordinators and
2. results from a qualitative evaluation of the project [22], [23], in which participants were interviewed regarding their professional role concepts and perceived changes due to the course.

By combining these two sources, we identified attitude dimensions that were considered relevant by both, IPE experts and IPE recipients. Different from other IPE instruments we, thus, took into account the perspective of the target group at an early stage of instrument design. 

The course coordinators identified two basic attitudes: “Attitude towards another profession” (e.g. “I find it important for my work to respect the opinion of the other profession”) and “attitudes towards interprofessional communication”. Based on a pilot study (n=213) the latter dimension was split up into: “Benefits of interprofessional communication” and “Relevance of learning interprofessional communicational techniques”. As a result of the qualitative interviews, we included two additional dimensions: “Willingness to cooperate in an interprofessional team” and “Doubts, dismissal and perceived barriers”.

#### 1.5. Phrasing of questionnaire items

The better the wording of the items support the cognitive processes underlying the answer, the higher the validity of the measurement. To answer an item a person must:

1. Understand the question,
2. access the relevant information in the memory,
3. estimate the frequency/intensity,
4. map the response to the response format and,
5. edit the answer for reasons of social desirability (see [11]).

Thus, to answer an item such as "individuals in other professions think highly of my profession" [24] a person must mentally calculate something like a mean, by mentally integrating probable judgements of different occupational groups that fall under "other professions". Whereby the perception of these groups may differ depending on the nature and frequency of professional contact [25]. Furthermore, instead of asking about the attitude of the person answering the questionnaire, a judgement about the thoughts of a nonspecific group of “others” is required. Even though this makes it hard to give a reasonable answer, respondents still try to do so by interpreting the question until it makes sense to them. However, due to different interpretations, similar numerical scores may then express dissimilar levels of attitude. 

IPE evaluation is further complicated by the fact that members of different professional groups are supposed to interpret and answer the items in the same way. Which in the case of an item like "I have to acquire much more knowledge and skills than other health care students" [25] seems rather unlikely, e.g. when comparing doctors and nurses. 

#### 1.6. Sensitivity to change

For a valid comparison of pre-post measurements, an instrument has to be sufficiently change-sensitive. Therefore, the instrument should not be too easy or too difficult in the tested sample [26]. All – or at least most – respondents agree with an easy item and reject a difficult one. However, when almost all respondents give the same answer, it is not possible to differentiate between respondents. Furthermore, identical answers lead to low variances of the test scores, which in turn impairs the calculation of scores based on the variance, such as reliability. Further pre-requirements for change sensitivity are the measurement of dimensions that actually can be changed by IPE courses, but still have sufficient stability. These criteria are met by attitudes. However, for the attitude changes to be meaningful, it must be demonstrated that they do not vary randomly. Thus, a minimum requirement is that without participating in an IPE course the attitudes remain somewhat stable.

## 2. Development of the questionnaire

### 2.1. Wording of the items 

The wording of the items was based on the following guidelines derived from the methodological arguments discussed above:

1. The items ask about personal attitudes and perceptions that can be measured validly via self-reports, and
2. are phrased in the first person if possible.
3. The wording aims to promote an identical interpretation of the items for different professional groups.

As response format we applied a five-point scale, ranging from 0 to 100 (0, 25, 50, 75, 100), with 0=disagree completely and 100=fully agree. The equidistance of the numerical scale values is visualized by the graphic representation of the scale (see [27]).

#### 2.2. Consistent interpretation

To foster a consistent interpretation of the items by different health care professions, explanations of relevant terms were provided in the instruction: “The questions address the cooperation between different health care professions. Thus, the term “interprofessional communication” describes the communication between these different professions”. 

Moreover, to avoid heterogeneous interpretations of items, the professional group addressed in the items was explicitly stated in the instruction. As the GreiFMIE, at this stage of development, was applied to evaluate courses for pre-qualification health professional students (mostly doctors and nurses), the instructions read as follows: “The term “members of the other occupational group”, refers to persons who are not members of your own professional group: i.e. nursing trainees if you study medicine and medical students if you are trained to be a nurse”. This was followed by items such as “Members of the other occupational group are usually competent”. The instruction has to be adapted depending on the professional groups (e.g. speech therapist or doctors) attending an IPE course. 

#### 2.3. Method

At the Phillips University of Marburg an IPE course “Mutual respect – care in a team” [28] was evaluated with the GreiFMIE. We were provided with the data for the development of the questionnaire. Consequently, we analysed data from two different samples, one from Greifswald (n=266) and one from Marburg (n=78). In Greifswald, the questionnaire was randomly applied either before or after participants attended the IPE course. By contrast, in Marburg participants filled out the questionnaire before and after the course. For the analysis, we randomly chose the pre or the post values of a participant. In both the cities, the courses were mostly attended by medical students and nursing trainees. Thus, in the first step of the analysis, we focus on these two professional groups.

#### 2.4. Sample

A description of the samples is depicted in table 1 (Tab. 1). 

## 3. Results

### 3.1. Factorial validity

An exploratory factorial analysis provides information about the structure underlying a set of items. Here, the set of items developed to represent the five attitude dimensions. We conducted a Principal Axis Analysis, followed by a Promax-Rotation because we expected the five attitudes dimensions to correlate. This yielded a five-factor solution (five factors with an eigenvalue>1), explaining 53% of the variance. However, the factors only partly matched the a-priori identified attitude dimensions: The dimension “Attitude towards another profession” was completely reproduced. All items a-priori designed to represent this factor, showed substantial loadings (see table 2 (Tab. 2)). By contrast, the factors “Relevance of learning interprofessional communicational techniques” and “Doubts, dismissal and perceived barriers” were only partially replicated. However, for each dimension we had more items drafted than we planned to include in the final instrument, thus, items that did not load on the respective factors were eliminated. Finally, for the two factors “Benefits of interprofessional communication” and “Willingness to cooperate in an interprofessional team”, no consistent structure of the items were found. Thus, the internal structure of the questionnaire was only partially replicated. 

Following the standards proposed by Oates and Davidson [7], we next calculated Cronbach’s α. The value indicates the internal consistency of a scale. The acceptable range of values depends on the area of application and ranges from .7 to .9 [29]. For the scales “Relevance of learning interprofessional communicational techniques” (α=.85) and “Doubts, dismissal and perceived barriers” (α=.73) the obtained Cronbach’s α fell within these limits, while the value for “Attitude towards another profession” did not meet the criteria (α=.62). This might be due to the highly positive attitudes expressed by the participants. Three items of this scale had a mean of M≥90 and a standard deviation of SD≤13.5, and were thus too easy in the tested sample.

#### 3.2. Stability

To test the stability and re-test reliability of the scales, 14 nursing trainees (age: M=20, SD=5.28, gender: w=11) completed the questionnaire twice within a span of 2.5-3 months. The trainees did not participate in any IPE training during this time. For the scales “Attitude towards another profession” (r_tt_=.73) and “Doubts, dismissal and perceived barriers” (r_tt_=.75) the re-test reliability was good. By contrast, the score for the scale “Relevance of learning interprofessional communicational techniques” was insufficient (r_tt_=-.18). However, once again, agreement with the items were extremely high and consequently the variance extremely low. At the first point of measurement, the modal was typically 100. Thus, this scale was far too easy for the tested sample.

## 4. Discussion

The GreifMIE aims to reliably and validly assess attitudes that

1. foster interprofessional cooperation
2. and can be influenced by IPE-courses.

Throughout its development, we sought to minimize psychometric problems typical for IPE-evaluation. At this point, there is no consistent theory of IPE [30], based on which different IPE courses are designed. Nevertheless, the development of the GreiFMIE was guided by theory because

1. the choice of the measured outcome level was driven by theory and
2. we applied a rational/deductive strategy of questionnaire development [31].

Based on expert knowledge and the perceptions of IPE recipients, we proposed various assumptions about the nature and structure of relevant attitudes, and then followed psychometric rules for the wording of the items, representing the attitude dimensions (see [32]). Finally, we provided precise instructions for the different scales and professional groups.

Via factor analyses, three of the five postulated attitude dimensions were replicated. Whereby two showed good internal consistencies and two good retest reliabilities. Furthermore, the Cronbach’s α of the scale “Attitude towards another profession” is with α=.64 still above the values of some scales applied in German-speaking countries (see [30]). The main reason for insufficient reliabilities seems to be that some items were not difficult enough. This may be a consequence of the sample because young people in health care education are often open-minded, and hence have very positive attitudes. The items may be more difficult in a sample in which the age, as well as the duration of employment of the participants are more diverse.

The scale “Doubts, dismissal and perceived barriers” introduces an aspect that may influence the willingness to learn and apply interprofessional skills considerably. Naturally, an IPE course will not eliminate professional hierarchies within a healthcare system. However, a change in this attitude dimension might foster successful cooperation despite obstacles at the work-place. Consequently, the GreiFMIE allows for testing hypothesis regarding different types of course concepts, because based on the respective concept it can be predicted which of the attitude dimensions are expected to change. Reeves & Hean [33] discuss the so-called "contact hypothesis" [34] as a theoretical explanation of attitudinal changes by IPE courses. Through the contact of the members of different groups, the attitude of the group members towards each becomes more positive. However, for this to happen, certain pre-requirements must be met (see [35], p. 18). Thus, an IPE course meeting these requirements are expected to show more positive attitudes towards another profession, but maybe not change the perception of the obstacles of interprofessional cooperation in everyday working life.

### 4.1. Limitations

So far, the results are based on data of only two occupational groups (medical students and nursing trainees), while results for other health care professions are still pending. However, for the evaluation of courses with a more divergent group of professionals, the psychometric quality of the questionnaire must first be determined for these groups.

Moreover, the scales have to be complemented with more difficult items. For this, we first need empirical evidence on whether the difficulty of the items depends on the age and duration of employment or other features of the respondents and also data regarding the re-test-reliability. Therefore, additional research with large and heterogeneous samples is necessary. 

The internal structure of the questionnaire has been confirmed, only partially. We cannot yet measure a core dimension such as “Benefits of interprofessional communication” validly. Currently, new items are being developed based on interviews with members of different health professions. Another key aspect, especially for the comparison of professional groups, is the so-called measurement invariance [36], which cannot be validly tested yet at this point of questionnaire development.

#### 4.2. Conclusion

Despite the limitations, The GreiFMIE offers a promising, also at this point somewhat provisional instrument for the measurement of IPE specific attitudes. The psychometric requirements of IPE evaluation instruments were explicitly addressed and empirically tested during the development of the questionnaire. Problems, such as insufficient difficult items or the valid assessment of the dimension Benefits of interprofessional communication, are currently addressed and will be eliminated in the next steps of the development.

## Funding

We acknowledge support for the Article Processing Charge from the German Research Foundation (DFG, 393148499) and the Open Access Publication Fund of the University of Greifswald.

## Competing interests

The authors declare that they have no competing interests. 

## Figures and Tables

**Table 1:**
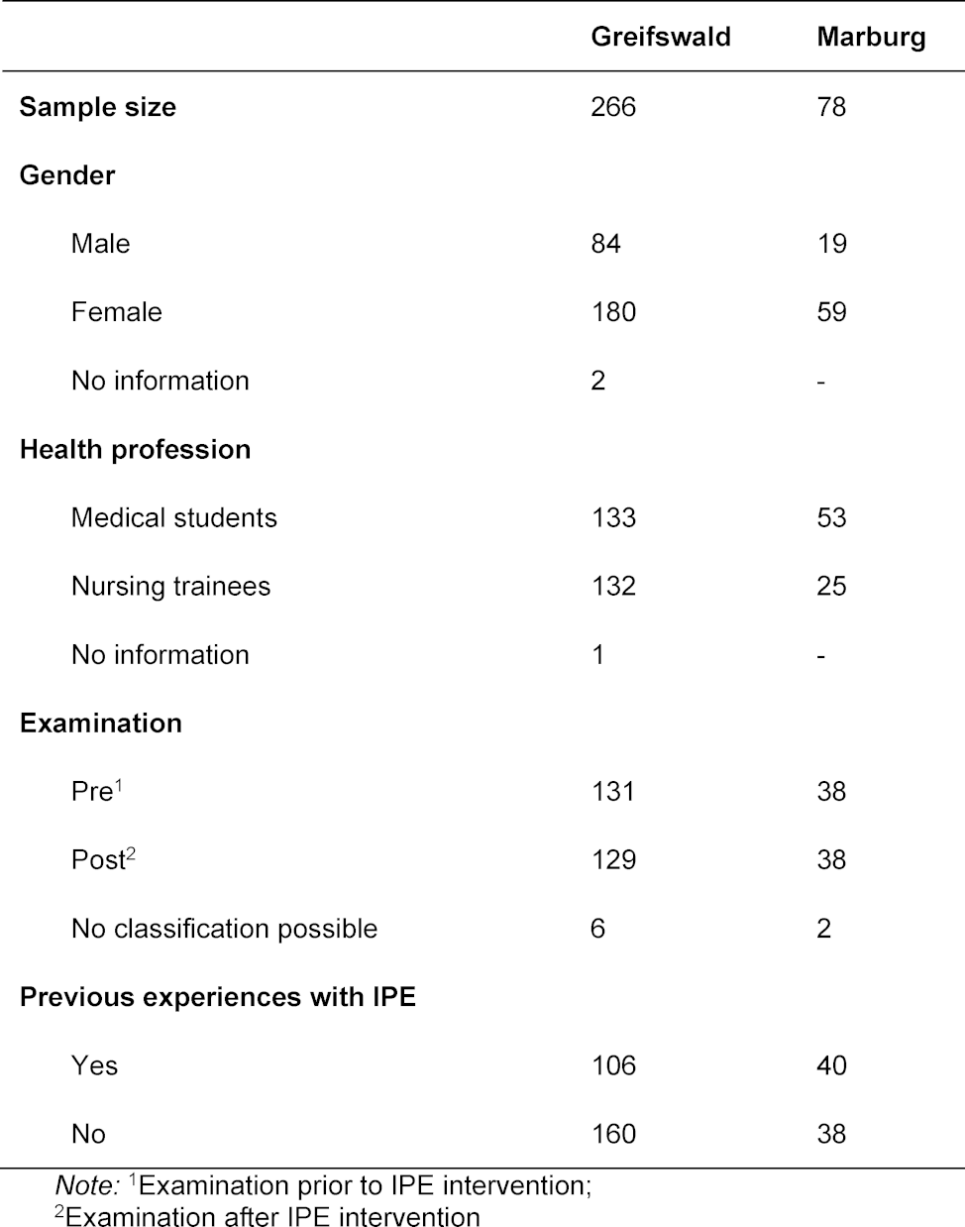
Sample description.

**Table 2:**
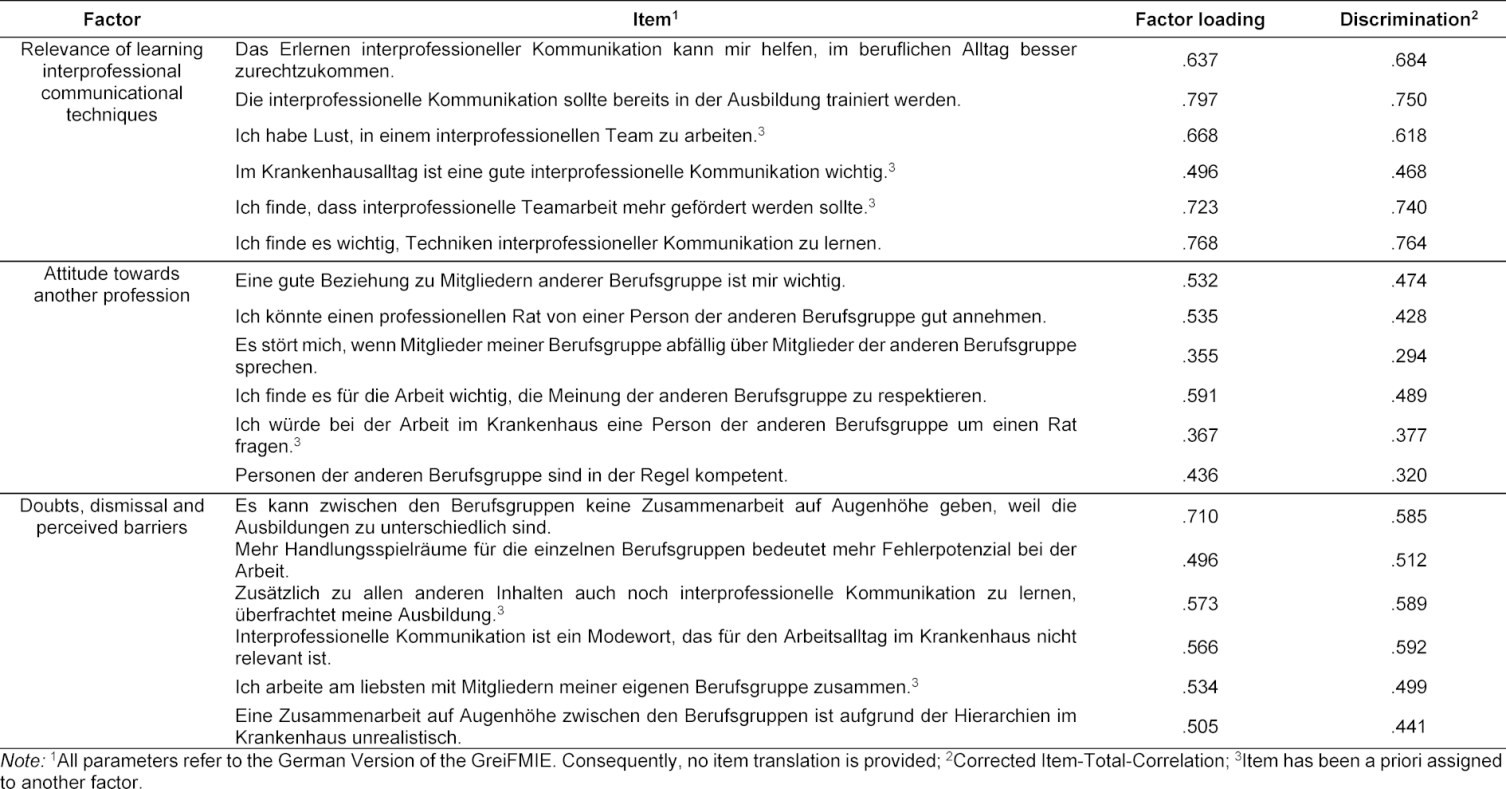
Factor loadings and item discriminations of the three replicated GreiFMIE factors.
